# Genetic analysis of complement factor H related 5, *CFHR5*, in patients with age-related macular degeneration

**Published:** 2009-04-10

**Authors:** Umadevi Narendra, Gayle J.T. Pauer, Stephanie A. Hagstrom

**Affiliations:** 1 Department of Ophthalmic Research, Cole Eye Institute, Cleveland Clinic Foundation, Cleveland, OH; 2 Department of Ophthalmology, Cleveland Clinic Lerner College of Medicine of Case Western Reserve University, Cleveland, OH

## Abstract

**Purpose:**

To investigate the complement factor H related 5 (*CFHR5*) gene, encoding a member of the complement factor H family, for the presence of genetic polymorphisms or mutations associated with age-related macular degeneration (AMD).

**Methods:**

We screened 639 unrelated patients with AMD and 663 age-matched normal controls using direct genomic sequencing of the ten coding exons, along with the immediately flanking intronic DNA. The pathologic impact of the identified sequence variants were analyzed by computational methods using PolyPhen and PMut algorithms.

**Results:**

We identified five heterozygous sequence changes in *CFHR5*. Asp169Asp had a minor allele frequency of 0.001% in patients and 0.014% in controls (p<0.0001), while Arg356His had a minor allele frequency of 0.016% in patients and 0.007% in controls. Val379Leu, Met514Arg, and Cys568Ter were found only in normal controls. In silico analysis predicted Arg356His and Val379Leu to be neutral and benign. Met514Arg was predicted to be pathological and damaging to the function of the CFHR5 protein.

**Conclusions:**

No definitive pathogenic *CFHR5* mutations have been found in any of 639 unrelated patients with AMD, indicating that sequence variations in *CFHR5* do not play a major role in determining AMD susceptibility. However, our findings suggest a possible protective role for Asp169Asp. Further studies of different and larger populations of patient and control samples will be required to address this observation.

## Introduction

Age-related macular degeneration (AMD) is the leading cause of irreversible visual loss in the elderly Western population over the age of 60 [1]. AMD is a progressive disorder of the photoreceptor-retinal pigment epithelium (RPE)-Bruch’s membrane-choriocapillaris complex. AMD typically starts with a few macular drusen, the lipoproteinaceous extracellular deposits localized between the RPE and Bruch’s membrane, which is considered to be a major risk factor for developing the disease. However, continued drusen evolution can lead to geographic atrophy, choroidal neovascularization, and irreversible vision loss in the later stages [1]. AMD is a complex, multifactorial disease associated with environmental, dietary, and genetic factors. Substantial evidence indicates that AMD has a strong genetic component, including the recent discoveries of polymorphisms in genes involved in the regulation of the immune-mediated complement pathway including complement factor H (CFH), complement factor B, complement component 2, and complement component 3 (C3) [2–7]. The nonsynonymous C versus T nucleotide polymorphism in the *CFH* gene, substituting histidine for tyrosine at position 402 (Y402H), has been identified as the most significant risk factor for developing AMD.

**Figure 1:**
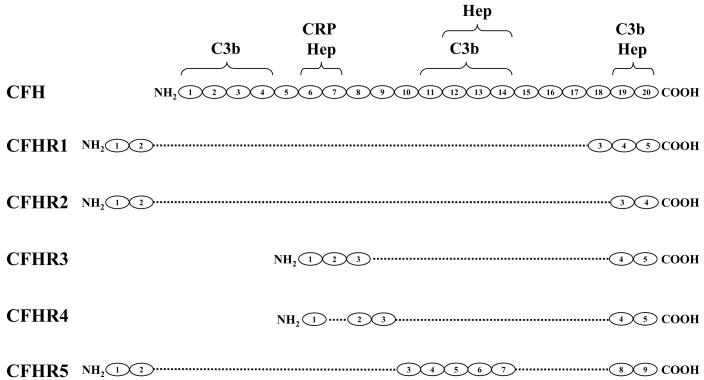
Schematic representation of the genomic structures of the genes in the *CFH* gene family. The CFH family is composed of several distinct proteins that lie in a gene cluster on chromosome 1. Structurally, these proteins are similar. They each contain several functional domains called short consensus repeats (SCRs), which are drawn as ovals in this illustration. The interacting proteins for some of these SCRs have been determined and are shown at the top. Abbreviations: C reactive protein (CRP); heparin (Hep).

Several regulators of complement activation, including CFH, lie in a gene cluster and have been mapped to chromosome 1q32. An evaluation of AMD genome-wide scans reveals that the strongest linkage to AMD is on chromosome 1q25–32 [8]. This array includes genes that encode the seven proteins in the CFH family (Figure 1). Structurally, these proteins are similar, each being built on a motif of distinct functional domains typical of the regulators of complement activation called short consensus repeats (SCRs). The interacting partners with some of these SCRs include C reactive protein (CRP), C3b, and heparin [9]. CFH-like 1 (CFHL1) is a splice isoform of CFH, while complement factor H-related proteins 1–5 (CFHR1–5) are each encoded by a unique gene (*CFHR1–5*). The SCRs of CFHR1–5 are similar to some of the SCRs in CFH [10].

Within the CFH family, the most recently discovered component is CFHR5. CFHR5, which shows the highest similarity to CFH, has nine SCRs, with the first two being similar to SCRs 6 and 7 of CFH and therefore having CRP and heparin binding properties [10]. SCRs 3–7 of CFHR5 are homologous to SCRs 10–14 of CFH, and SCRs 8–9 show homology to the C3b binding domain of CFH in SCRs 19 and 20. CFHR5 is the only CFHR protein that, like CFH, possesses cofactor activity leading to the inactivation of C3b. CFHR5 binds to human C3b in a dose-dependent manner [11–13]. The complement regulatory activity of CFHR5 has been proposed to rely upon its recruitment to sites of tissue damage by CRP [12].

As mentioned, several studies have implicated specific allele variants of *CFH* with the development of AMD. In addition, a replication study on the NEI dbGAP single nucleotide polymorphisms (SNPs) derived from the genome-wide association study on the Age-Related Eye Disease Study (AREDS) patient cohort demonstrates that many variants located in tandem on 1q32, including several in *CFH*, *CFHR2*, *CFHR4,* and *CFHR5*, were significantly associated with AMD risk [14].

Risk alleles in *CFH* have also been reported in patients with two different forms of kidney disease, membranoproliferative glomerulonephritis type II (MPGN II), and hemolytic uraemic syndrome (HUS) [10,15]. MPGN II is a renal disease characterized by mesangial hypercellularity and an accumulation of electron-dense material in the lamina densa of the glomerular basement membrane. MPGN II causes chronic renal dysfunction that can progress to end stage renal disease. HUS is a rare syndrome characterized by the triad of microangiopathic hemolytic anemia, thrombocytopenia, and acute renal failure. It is the most common cause of acute renal failure in children. Patients with MPGN II, HUS, and AMD segregate several of the same *CFH* risk alleles [10,15]. Patients with MPGN II also develop ocular drusen that are clinically indistinguishable from drusen that form in AMD [16,17]. The sole difference between these two types of drusen is the age of onset; drusen in MPGN II develop early around the second decade of life, whereas drusen in AMD are found in the fifth or sixth decade of life. Moreover, cases have been described where MPGN II patients develop AMD later in life [18]. Recently, SNPs in *CFHR5* have been associated with both MPGN II and HUS, suggesting an important role for CFHR5 in the protection of cells against complement activation at least in the glomeruli of kidneys, although its exact functional properties remain unknown [10,15].

The possible relationship between MPGN II and AMD as diseases that share a genetic association with complement genes as well as complement proteins characteristic of their dense deposits motivated us to hypothesize that like CFH, CFHR5 could predispose to the development of AMD. Furthermore, recent evidence indicates that variants in *CFHR* genes may confer risk for AMD. To evaluate this possibility, we screened a large number of AMD cases and age-matched controls for genetic abnormalities in *CFHR5* that are associated with AMD.

## Methods

### Ascertainment of patients

This study conformed to the tenets of the Declaration of Helsinki and was approved by the Internal Review Boards of the Cleveland Clinic Foundation. All blood samples were obtained after informed consent was secured. We collected a total of 639 index cases with AMD that were diagnosed through ophthalmologic examination and were subcategorized into one of four experimental groups using the criteria defined by the AREDS study [19]. Briefly, AMD Category 1 subjects had five or less small drusen/eye (<63 µm) and visual acuity of 20/30 or better in both eyes. AMD Category 2 patients exhibited early-stage disease with multiple small drusen, single or nonextensive intermediate drusen (63–124 μm), RPE pigmentary abnormalities, or any combination of these, in one or both eyes and visual acuity of 20/30 or better in both eyes. AMD Category 3 patients exhibited mid-stage disease with at least one eye having visual acuity 20/30 or better and one large drusen (125 μm), extensive intermediate drusen or geographic atrophy that did not involve the macula or any combination of these. Category 3 patients lacked advanced AMD in either eye. AMD Category 4 patients exhibited advanced AMD with substantial choroidal neovascularization or geographic atrophy involving the macula in one or both eyes.

Of the 639 patients, 26 were defined as Category 1 AMD, 43 were Category 2, 90 were Category 3 and 480 were Category 4. AMD Category 1 consisted of 12 males and 14 females. AMD Category 2 consisted of 12 males and 31 females. AMD Category 3 consisted of 35 males and 55 females and AMD Category 4 consisted of 88 males and 292 females. 629 patients were over the age of 60 and the remaining 10 were over the age of 53. The age range was between 53 and 105 with a mean age of 81.7.

A total of 663 unrelated individuals without retinal disease as diagnosed by ophthalmologic examination and without a family history of retinal disease were used as normal control subjects. In our control population, there were 298 males and 365 females. The age range was between 50 and 103 with a mean age of 70.3. Leukocyte nuclei were prepared from the blood samples followed by DNA purification using standard protocols.

### Mutation screening

For mutation detection, PCR products corresponding to the complete known *CFHR5* coding sequence (accession number BC111773) were amplified from genomic DNA and analyzed by direct genomic sequencing. Ten primer pairs were designed to cover the ten coding exons and adjacent intronic regions of *CFHR5* and are listed in Table 1 along with the PCR conditions. The buffer pH, Mg^2+^ concentration, annealing temperature, and presence or absence of 10% dimethyl sulfoxide were tailored to each primer pair to yield optimal amplification.

**Table 1:** PCR conditions and primers used to amplify *CFHR5* in this study.

**Exon**	**Primers (3′-5′)**	**Annealing temperature (°C)**	**Buffer pH**	**MgCl_2_ (mM)**	**% DMSO**	**Fragment (bp)**
1	F: GCAGATTTAAAGCAACACCACC	60	8.4	1	0	242
	R: CCCCTTCAAATTATCCTCAGC					
2	F: CTAGTGATTCATCGATGTAGCTC	52	8.4	1	10	370
	R: TTTCCAGCTCCTCTGGTCATTGC					
3	F: TGATGTCAGTTTTCAAAGTTTTCC	54	8.4	1	0	313
	R: TAATTAGCAAAACTGAGAGAGTGG					
4	F: GCACACATTAAATTTGTTTCTGCA	54	8.4	1.5	0	345
	R: TACCACTTTGTCAGATTATAGATG					
5	F: TAGTAACTCCACATTTTTCTATAC	50	8.4	1.5	0	301
	R: TAAGTGCAAAGTAATAGTAACTGT					
6	F: AATATTTTCAGAGTAAGCACTCAT	50	8.4	1.5	0	288
	R: ACACTTTATACAATCCCAATCAAA					
7	F: GTGATGTTGCTTAAAAGCATCAAAC	54	8.6	1.5	0	325
	R: CTTGTAAAGAAGCAACAAGATCAAC					
8	F: CCATTTTCCTGAAACACTACCCTA	52	8.4	1.5	0	377
	R: TTGGGGTACAGTGCAACAGATTAG					
9	F: AATTATTTGAATTTCCAGACACCT	50	8.4	1.5	0	375
	R: GGGTTATTCTATGAAATTAGTCCA					
10	F: GCAATTTCACTATTCTATGAAAGG	52	8.4	1.5	0	348
	R: ACAGAATTGGCTACATAATGGCT					

This table summarizes the PCR conditions and primers used to amplify *CFHR5* in this study.

Unused primer molecules and dNTPs were removed from the amplified PCR product by treating with ExoSAP-IT (USB, Cleveland, OH) at 37 °C for 15 min and at 80 °C for 15 min to inactivate the enzymes. Sequencing was performed using 2 μl of BigDye Terminator v1.1 cycle Ready Reaction Mix (Applied Biosystems, Foster City, CA), 3 μl of BigDye Terminator v1.1 cycle Sequencing Buffer, 5 pmol of sequencing primer and 5 μl of ExoSAP-IT purified PCR product in a total volume of 20 μl. Cycling parameters followed the manufacturer’s instructions. Unincorporated dye terminators were removed using Performa DTR gel filtration plates (Edge Biosystems, Gaithersburg, MD). Sequencing was performed using an ABI 3130xl Genetic Analyzer (Applied Biosystems), and the collected data was analyzed with SeqScape v2.5 software.

### Computational assessment of missense mutations

Two sequence homology based programs were used to predict the functional impact of missense changes identified in this study: PolyPhen (Polymorphism Phenotyping,) and PMut. PolyPhen structurally analyzes an amino acid polymorphism and predicts whether that amino acid change is likely to be deleterious to protein function [20]. The prediction is based on the position-specific independent counts (PSIC) score derived from multiple sequence alignments of observations. PolyPhen scores of >2.0 indicate the polymorphism is probably damaging to protein function. Scores of 1.5–2.0 are possibly damaging, and scores of <1.5 are likely benign. PMut allows the accurate pathological prediction of single amino acid mutations based on the use of neural networks [21]. Following the input of a reference sequence and the amino acid substitution of interest, the algorithm provides an answer and a reliability index. An output value >0.5 is predicted to be a pathological mutation and a value <0.5 is neutral. The reliability is considered good with a score of six and greater and is highly reliable at the maximum score of nine.

### Statistical analysis

The χ^2^ test was used to analyze Hardy–Weinberg equilibrium. A two-tailed Fisher’s exact test was used for comparisons of allele frequencies.

## Results and Discussion

In the present study, we analyzed the *CFHR5* gene for mutations in patients with AMD to search for sequence changes that might be associated with disease. Because of the strong homology between CFH and CFHR5, the possible relationship between MPGN II and AMD as diseases that share a genetic association with complement genes, and recent evidence of variants in CFHR genes that may predispose an individual to AMD, we considered this gene a candidate for AMD.

Five SNPs in *CFHR5* have been associated with MPGN II, a renal disease that shares genetic association with genes associated with AMD [15]. These include one nonsynonymous SNP (exon 2 P46S), two promoter SNPs (−249T→C and −20T→C), and two intronic SNPs (IVS1+75T→A and IVS2+58C→T). Allele frequencies of three (exon 2 P46S, −249T→C and −20T→C) were significantly different between patients and controls. We postulate that the two SNPs in the promoter region could affect transcription, one by removing a binding site for C/EBPβ and the other by adding a GATA-1 binding site. The missense change proline to serine in exon 2 encodes a domain homologous to SCR6 of CFH, which is integral to heparin and C reactive protein binding, therefore possibly affecting complement activation and control. Our mutation analysis screened for all five of these SNPs in 639 patients with AMD and did not identify any of these genetic changes in our patient population.

Five *CFHR5* variants different from those described in MPGN II patients have been associated with HUS, a renal disease that also shares genetic association with AMD [10]. These variants include four missense changes (Leu66Phe, Lys126Asn, Arg338His, and Met496Arg) and one stop mutation (Asn197Ter). Our mutation analysis screened for all five variants in 639 patients with AMD and did not identify any of these genetic changes.

Instead, we identified five different heterozygous sequence changes in *CFHR5* (Table 2); however, none appear to be definitively associated with disease. No significant deviations from Hardy–Weinberg equilibrium for any of the variants studied were observed. Two variants (Asp169Asp and Arg356His) were identified in AMD patients and normal controls while the other three (Val379Leu, Met514Arg, and Cys568Ter) were found only in controls.

**Table 2:** Sequence Alterations Identified in *CFHR5.*

**Exon**	**Sequence change**	**Protein change**	**Allele frequency AMD patients**	**Allele frequency normals**	**p value**
4	GAC→GAT	Asp169Asp	1/1278=0.001%	18/1326=0.014%	p<0.0001
7	CGT→CAT	Arg356His	20/1278=0.016%	9/1326=0.007%	p=0.039
7	GTA→CTA	Val379Leu	0/1278=0.0%	3/1326=0.002%	p=0.250
10	ATG→AGG	Met514Arg	0/1278=0.0%	1/1326=0.001%	p=1.000
10	TGT→TGA	Cys568Ter	0/1278=0.0%	3/1326=0.002%	p=0.250

This table summarizes the sequence changes identified in *CFHR5* in this study.

The Asp169Asp synonymous SNP was identified in one Category 4 AMD patient, yielding a minor allele frequency of 0.001%. Asp169Asp had an allele frequency of 0.14% in normal controls and appeared to be a common variant as it is present in the NCBI SNP database (rs34533956). This isocoding change does not alter the predicted wild-type amino acid sequence nor is it predicted to create or destroy splice donor or acceptor sites based on splice-site prediction software by neural network available at the Berkeley Drosophila Genome Project; however, it warrants additional consideration. Our data indicate that the mutant T allele is less frequent in AMD patients as compared to normal controls (p<0.0001), suggesting that this variant is associated with a reduced risk for developing AMD. Whether this variant is a protective allele or is simply linked to a different nearby gene variant remains to be determined. If protective, it is possible that this sequence change exerts its effects by improving mRNA stability or by effecting the splicing of RNA transcripts which is thought to be partly regulated by sequences embedded within exons [22,23]. Silent polymorphisms within genes have been shown to influence the quality and fidelity of mRNA metabolism such as the rate of gene transcription, the stability of the mRNA, or the quantity and activity of the resulting protein [24]. Sequences known to affect RNA splicing include exonic splicing enhancers or exon splicing silencers, which consist of common motifs of only 6–8 bases and activate nearby splice sites and promote the inclusion or exclusion of exons in which they reside [25]. In addressing our observation, additional studies of different and larger populations of patient and control samples will be required as well as functional studies concerning this specific sequence change.

In exon 7, Arg356His was found in 20 patients: 15 from Category 4, two from Category 3, and three from Category 2. It was also identified in normal controls. The minor allele frequency for this sequence change is 0.016% in patients and 0.007% in normal controls (p=0.039). Arg356His also appears to be a common variant and is present in the NCBI SNP database (rs35662416). In silico analysis using PolyPhen predicted this variant to be benign. A small difference (1.190) was noted in PSIC scores between the allelic variants. PMut analysis also predicted Arg356His to be neutral (output value of 0.3) with good reliability (score of 5).

Two missense changes (Val379Leu and Met514Arg) and one stop mutation (Cys568Ter) were found heterozygously only in controls and not in any category of AMD patients. Val379Leu had a minor allele frequency of 0.002%. Both PolyPhen and PMut analysis predicted Val379Leu to be a neutral (output value of 0.3) and benign substitution with high reliability (score of 4). The rare variant Met514Arg warrants consideration. It was identified in one female control aged 79, yielding a minor allele frequency of 0.001%. Interestingly, PolyPhen predicted this missense change to be probably damaging. A large difference (2.852) was calculated in PSIC scores between the allelic variants. This difference indicates that the observed substitution is rarely or never observed in the CFH protein family and is predictive of a hydrophobicity charge change at a buried site within the protein. PMut analysis also predicted Met514Arg to be pathological (output value of 0.9) with very high reliability (score of 7). It is possible that this mutation affects the function of CFHR5 by changing the neutral, nonpolar side chain of methionine to the basic, positively charged side chain of arginine. In fact, the location of this mutation is embedded within the C-terminal region of the protein that binds to C3b and Heparin. The stop mutation Cys568Ter was identified in three normal control individuals aged 58, 61, and 85, yielding a minor allele frequency of 0.002%. This mutation occurs in the penultimate amino acid of the CFHR5 protein and therefore may not affect protein function.

Our results indicate that sequence variations in *CFHR5* are extremely rare in patients with AMD. Five sequence variations were identified with very low frequency. All are likely to be nonpathogenic polymorphisms as two are known SNPs and three were identified only in normal controls. The nonsynonymous missense changes (Arg356His and Val379Leu) were consistently estimated to be neutral or benign by computational analysis. However, the Met514Arg missense change warrants additional consideration. Both bioinformatic algorithms predicted this mutation to be pathological and damaging to the function of CFHR5. Although this mutation was identified in a single age-matched control individual, she should be evaluated by an ophthalmologist for the possible development of AMD. Whether this variant is pathological remains to be determined. Asp169Asp also deserves consideration. The frequency of the mutant allele was significantly higher in controls than in all AMD patients (p<0.0001), suggesting that this variant is acting to decrease susceptibility to AMD. In addressing these observations, further studies of different and larger populations of patient and control samples will be required.

It is possible that SNPs associated with AMD as well as pathogenic mutations in *CFHR5* may exist outside of the coding exons and flanking intron splice sites screened in this study. It is not unreasonable to consider noncoding DNA variants to underlie a complex disease phenotype, such as AMD, as complex conditions are likely to be caused by several common variants across a variety of loci, each responsible for only minor effects. These minor effects may be the result of DNA variants in the noncoding regulatory elements, which may be capable of subtle changes in gene expression in a tissue or developmental stage-specific manner. Finally, it is also possible that pathogenic mutations occur in *CFHR5* in a form of retinal degeneration not included in our patient set. Nonetheless, our results indicate that *CFHR5* mutations or polymorphisms are not definitively associated with AMD.
